# New Insights in Human Myocarditis

**DOI:** 10.3390/jcm11040924

**Published:** 2022-02-10

**Authors:** Andrea Frustaci

**Affiliations:** Department of Clinical, Internal, Anesthesiologist and Cardiovascular Sciences, La Sapienza University, Viale del Policlinico 155, 00161 Rome, Italy; biocard@inmi.it; Tel.: +39-06-5517-0520

Myocarditis is an inflammatory heart muscle disease, the incidence of which is likely underestimated, ranging from 10.2 to 105.6 per 100,000 individuals worldwide [1]. Although many tools may contribute to its recognition, such as ECG, echocardiogram, cardiac magnetic resonance (CRM) and elevated biomarkers such as Troponin I, the diagnostic gold standard remains endomyocardial biopsy (EMB) [2,3]. Indeed, in addition to histologic findings (the immuno-histochemical detection of ≥14 leukocytes/2 mm with damage to adjacent myocytes), the Dallas criteria [4] recognises specific inflammatory lesions such as eosinophilic, granulomatous and giant cell infiltrates that provide direct information regarding the underlying cause and the appropriate treatment to use. When infectious myocarditis is suspected, the application of polymerase chain reaction to frozen endomyocardial samples allows for the identification of the causative inflammatory agent and how to manage it. In this regard, viral genomes are the most commonly involved agents, and nowadays, a specific strategy for their treatment is available [5]. 

A clear advancement in the treatment of myocarditis has been reached with the confirmation of the effectiveness of immunosuppressive therapy in virus-negative inflammatory cardiomyopathy. The TIMIC trial [6] suggested that 88% of these patients respond to a combination of prednisone and azathioprine administered for six months vs. supportive therapy alone, with positive cardiac remodelling and remarkable (>10%) improvement in the LVEF. The pathway of the remaining 12% of nonresponders remains to be explored; metagenomics is actually applied to recognise potentially new infectious agents. 

Recently, new, unconventional forms of myocarditis have been recognised, which help the interpretation and treatment of unclear clinical manifestations. Blunt thoracic trauma may be followed by cardiac dilatation and dysfunction which, using endomyocardial biopsy, has been attributed to myocarditis following the release of immunogenic myosin, which responds to the moderate and limited administration of steroids [7].

Untreated and severe gout can be associated with myocardial inflammation and heart failure, with the deposition of amorphous urate crystals in myocardiocytes becoming a reversible source of inflammation and heart failure [8].

The mechanical and/or electrical deterioration of hypertrophic cardiomyopathy seems to be promoted, in histologic and molecular investigation, to an auto-reactive or viral myocarditis that can be cured or improved using appropriate treatment [9].

Finally, infiltrative-like cardiac amyloidosis [10], as well as storage disease such as Anderson–Fabry cardiomyopathy, can be overlapped by myocardial inflammation that concur significantly to the disease progression [11]. Its proper interpretation and treatment may positively change patients’ outcome.

Currently, COVID-19 infection as well as its mRNA vaccine have been strongly implicated in the generation of myocarditis, sometimes becoming a cause of patient impairment and death.

While in the first instance a virus-negative immunogenic pathway is recognised [12,13], in the second instance, a hypersensitivity myocarditis similar to a drug reaction with the release of cationic protein from crystalloids of eosinophils can be recognised and correctly counteracted.
